# Exposure to Metal Mixtures and Childhood Adiposity: An Examination of Periods of Heightened Susceptibility Between Gestation and Late Childhood

**DOI:** 10.1111/ijpo.70057

**Published:** 2025-09-14

**Authors:** Janice M. Y. Hu, Michael M. Borghese, Mandy Fisher, Joseph M. Braun, Katherine M. Morrison, Mark R. Palmert, Linda Booij, Constadina Panagiotopoulos, Jillian Ashley‐Martin

**Affiliations:** ^1^ Environmental Health Science and Research Bureau, Healthy Environments and Consumer Safety Branch Health Canada Ottawa Ontario Canada; ^2^ Department of Epidemiology Brown University Providence Rhode Island USA; ^3^ Department of Pediatrics McMaster University Hamilton Ontario Canada; ^4^ Division of Endocrinology, Hospital for Sick Children and Department of Pediatrics University of Toronto Toronto Ontario Canada; ^5^ Division of Endocrinology, Hospital for Sick Children and Department of Physiology University of Toronto Toronto Ontario Canada; ^6^ Department of Psychiatry and Douglas Mental Health University Institute McGill University Montreal Quebec Canada; ^7^ CHU Sainte‐Justine Azrieli Research Center Montreal Quebec Canada; ^8^ Department of Pediatrics University of British Columbia Vancouver British Columbia Canada

**Keywords:** arsenic, body mass index (BMI), childhood obesity, metal mixture, periods of heightened susceptibility, tree distributed lag mixture model, waist circumference

## Abstract

**Introduction:**

Childhood obesity is a public health concern. Studies have investigated the effects of metal mixtures on childhood obesity but none have identified periods of heightened susceptibility of exposure. We identified the periods by investigating the association of metal mixture, measured at four time points, with adiposity.

**Materials and Methods:**

Using data from the Maternal‐Infant Research on Environmental Chemicals Research Platform, we included 234 child–parent pairs. We measured whole blood metal concentrations during the first and third trimesters, early and late childhood. Outcomes were late childhood body mass index *z*‐score (zBMI), body fat percentage (%BF) and waist circumference *z*‐score (zWC). We used treed distributed lag mixture models (TDLMM) to investigate associations between metal mixture and adiposity. We also investigated associations using linear regression and conducted sex‐specific analysis.

**Results:**

Among females, arsenic was positively associated with zBMI and zWC. Regression results show that each doubling in third trimester arsenic concentrations was associated with 0.16 (95% CI: 0.02, 0.31) and 0.13 (95% CI: 0.01, 0.25) increase in zBMI and zWC, respectively. TDLMM results were similar but attenuated. We also observed negative associations between third trimester cadmium and zWC, null associations between other metals and adiposity and among males and no metal interactions.

**Conclusion:**

Third trimester is a period of heightened susceptibility to obesogenic effects of arsenic exposure in females.

## 1 Introduction

The prevalence of childhood obesity has increased in recent decades to levels of global public health concern. According to the National Institutes of Health, about 1 in 5 American children (19% or 41 million) are currently living with obesity [1]. In Canada, it was reported in 2015 that about 1 in 7 (14%) children between 6 and 11 years old had obesity [2]. By 2030, an estimated 254 million children worldwide will live with obesity [3].

Childhood obesity is associated with increased morbidity and premature mortality [3]. It not only affects children's immediate health, educational attainment and quality of life, but also increases their risk of developing serious chronic diseases, such as cardiovascular diseases, type 2 diabetes and certain cancers in adulthood [4]. Obesity is a multifactorial condition that cannot be fully explained by food intake and physical inactivity. It is also influenced by genetic predisposition and environmental factors [5]. A better understanding of the aetiology of childhood obesity is critical to preventing children from becoming overweight or developing obesity.

Epidemiological studies have indicated that gestational exposure to environmental chemicals at low levels that are assumed to be safe may alter growth patterns and induce weight gain and obesity risk among children [6, 7]. A review by Tinkov et al. [8] reported that adipose tissue may be a potential target of metal toxicity. Specifically, low level metal exposure may promote adipogenesis, which could manifest as obesity [8]. Exposure to mercury (Hg), cadmium (Cd), lead (Pb) and arsenic (As), as well as to mixtures of these metals, has been associated with both obesity and metabolic syndrome [8, 9, 10]. Metal toxicity may also depend on the timing of exposure. For example, chemical exposure during periods of heightened susceptibility, such as pregnancy or child development, can result in more pronounced health effects than exposure when rapid physiological change is not occurring [11]. We are not aware of any epidemiological study that has examined the susceptible periods for childhood obesity risk.

To address this gap, our study identified periods of heightened susceptibility by investigating the potential impacts of exposure to a mixture of five whole blood metals (As, Cd, Hg, Pb and manganese [Mn]) at multiple time points, extending from the first trimester of pregnancy to late childhood, on adiposity measures in a cohort of 234 Canadian children who participated in the Maternal‐Infant Research on Environmental Chemicals Endocrine (MIREC‐ENDO) follow‐up study. We also stratified all analyses and examined the potential modifying effects of child sex assigned at birth.

## 2 Materials and Methods

### 2.1 Study Population

Data were drawn from the Maternal‐Infant Research on Environmental Chemicals (MIREC) Research Platform. The MIREC cohort profile and detailed eligibility criteria have been previously reported [12, 13]. Briefly, MIREC is a pan‐Canadian cohort study (2008–2012) with the goals of obtaining Canadian biomonitoring data on pregnant women across 10 cities and examining associations between exposure to environmental chemicals and child health outcomes [12]. The participants provided questionnaire data and biological specimens during each trimester of pregnancy. Subsequent to the initial MIREC pregnancy cohort, participants from six of the original 10 sites were invited to participate in a follow‐up study (MIREC‐CD Plus, 2013–2015) on child development and exposure to metals during early childhood (aged 2–5) [13]. A subset of these participants was further recruited into a subsequent follow‐up study (MIREC‐ENDO, 2018–2021) designed to investigate associations between environmental chemicals and child metabolic health during late childhood (aged 7–9). In the present analysis, we included 234 singleton‐born MIREC children who had at least one of the adiposity measurements of interest measured during late childhood and had available data on blood metal concentrations during pregnancy and childhood (Figure S1).

### 2.2 Child Adiposity Measures

To assess adiposity in children, we examined body mass index (BMI), waist circumference (WC) and body fat percentage (%BF) measured in late childhood (aged 7–9). BMI, despite its inability to distinguish lean from fat mass, is the most commonly used proxy for adiposity [14]. WC provides an indication of the distribution of adipose tissue [15] and %BF is a main component of body composition and a more direct measure of adiposity [14, 16].

Trained research personnel measured children's weight with a calibrated scale (Seca model 874) and standing height with a calibrated stadiometer (Seca model 217). We computed children's BMI as ([weight in kg]/[height in meters])^2^ and calculated age and sex‐standardised BMI *z*‐scores (zBMI) using World Health Organization child growth standards [17]. WC was measured with a flexible measuring tape (Seca 201 Ergonomic) at the midpoint between the lowest rib and the iliac crest. We calculated age and sex‐standardised WC *z*‐scores (zWC) based on the Canadian Health Measures Survey percentiles [18]. %BF was measured using a bioelectric impedance scale (Tanita SC‐240) [14]. %BF was not converted to *z*‐score because reference data are unavailable. For all three adiposity measures, two measurements were taken. In cases where discrepancies were greater than a predetermined value (i.e., weight at 0.1 kg, height at 0.5 cm and WC at 0.3 cm), a third measure was taken and the average of the two closest measures were used. All three measures were normally distributed and analysed as continuous variables.

### 2.3 Metal Exposure Assessment

To account for the potential time‐dependent nature of obesogens on children's health, we assessed five whole blood metal concentrations (As, Cd, Hg, Pb and Mn) at four time points: first trimester, third trimester, early childhood (aged 2–5) and late childhood (aged 7–9) (Figure S2). The Toxicology Laboratory of the Institut National de Santé Publique du Québec (Canada) performed the laboratory analysis for all time points. The analysis methods for samples collected during the first trimester, third trimester and early childhood have been previously described [19, 20] and the analytical method for samples collected during late childhood is described in Appendix A in Supporting Information.

We replaced prenatal concentrations below the limit of detection (< LOD) using the single imputation ‘fill‐in’ approach where the concentrations < LOD were randomly sampled from a truncated lognormal distribution with mean and standard deviation estimated from the observed data [21]. Machine reading data were used for concentrations < LOD during early and late childhood. Measurements with zeroes or negative values were replaced with the minimum positive value divided by 2.

To reduce the potential influence of statistical outliers due to the right‐skewed distributions of metal concentrations, the concentrations were log_2_‐transformed before inclusion in statistical models. The transformed unit indicates a two‐fold increase in concentrations.

### 2.4 Covariates

We created a directed acyclic graph (Figure S3) to identify determinants of childhood adiposity and metal exposure at all time points. The following covariates were included in all models: maternal age during pregnancy (< 30, ≥ 30 to < 35, ≥ 35 years), race and ethnicity (white, non‐white), birth country (Canada, foreign), education (college or trade school diploma or less, undergraduate university degree, graduate university degree), marital status during pregnancy (married, not married), cigarette smoking status during pregnancy (never, ever), parity (0, 1, ≥ 2), maternal pre‐pregnancy BMI (< 25.0, 25.0–29.9, ≥ 30.0 kg/m^2^), child's accelerometer‐measured moderate‐to‐vigorous physical activity level (average minutes per day) and clinical sites. We additionally adjusted the %BF models for child age at late childhood (continuous) and child sex assigned at birth (male, female). All covariates were complete with no missing values except for pre‐pregnancy BMI (*n* = 13) and child's physical activity (*n* = 93).

### 2.5 Analytical Approach

We tabulated participant characteristics, examined descriptive statistics for all adiposity measures, calculated geometric means and percentiles of the metals and examined the intraclass correlation (ICC) of the metals across time points [22]. We interpreted ICCs of ≤ 0.4 as poor, 0.40–0.75 as fair to good and ≥ 0.75 as excellent [23].

We used both conventional multivariable linear regression (MLR) and a novel mixture method to investigate the associations between metals and child measures of adiposity at multiple time points. To assess the mixture of the five metals, we applied a treed distributed lag mixture model (TDLMM) to examine main effects, relative importance of each metal within the mixture and pairwise interactions of the exposures observed at multiple time points. TDLMM is a tree‐based mixture model adapted from the Bayesian additive regression trees framework [24]. It uses a set of regression tree‐pairs to structure exposure measurements and estimate the main effects and pairwise interactions within a time point and across time points. TDLMM performs variable selection and shrinkage to remove any chemicals and interactions that are not associated with the outcome using posterior inclusion probabilities (PIP) to identify and select chemicals and pairwise interaction effects [24]. PIPs range from 0 to 1, with higher PIPs indicating greater variable importance. We identified important metals and interactions based on PIPs > 0.50. TDLMM effect estimates describe the difference in mean adiposity measures (i.e., zBMI, zWC and %BF) associated with a two‐fold increase in metal concentrations, adjusted for the same metal at other time points, other metals at all time points, pairwise interactions and covariates, while holding everything at their respective mean. We used the prior specification as described in Mork and Wilson [24] and ran the models for 50 000 iterations after 5000 burn‐in and thinned it to every 5th iteration.

We used MLR to examine the associations between individual metal concentrations and adiposity measures at each time point for those metals chosen by TDLMM and compared the results. The resulting beta coefficient describes the difference in mean adiposity measures associated with a two‐fold increase in metal concentrations, adjusted for covariates.

Prior to analysis using TDLMM and MLR, we imputed missing exposure and covariate data for participants with at least one prenatal and one postnatal observation using multiple imputation by chained equations (MICE) with 20 multiply imputed datasets (Appendix B in Supporting Information) [25, 26]. We also stratified all analyses and examined the potential modifying effects of child sex assigned at birth. Furthermore, we used residual plots to check for violations of model assumptions. All analyses were conducted using R 4.2.1 [27]. Missing observations were imputed using the *mice* package [26] and TDLMM models were fitted using the *dlmtree* package [24].

## 3 Results

### 3.1 Descriptive Statistics

We included a total of 234, 233 and 220 children and their birthing parents for analyses of zBMI, zWC and %BF, respectively. The mean (SD) child age when the adiposity measures were obtained was 8.9 (0.8). The majority of children were female (57%) and the children were physically active, on average, for over 60 min per day. The majority of parents were white (92%), born in Canada (88%), married (68%), over 30 years of age (69%), with at least an undergraduate degree or higher (70%), never smoked (68%) and had a pre‐pregnancy BMI < 25.0 kg/m^2^ (63%). Furthermore, 44% were nulliparous. Pregnant individuals with the following characteristics tended to have children with higher childhood zBMI: white, between the ages of 30 and 34, married, smoking, born in Canada, multiparous and with pre‐pregnancy BMI ≥ 30.0 kg/m^2^. The same characteristics, with the addition of higher educational attainment, were associated with higher zWC. The characteristics associated with higher %BF differed slightly and included being non‐white, older, lower educational attainment, married, smoking, born in Canada, multiparous and higher pre‐pregnancy BMI. Male children, on average, had higher zBMI but lower zWC and %BF, compared to female children (Table 1). Participant characteristics were comparable after imputation (Appendix C Table A in Supporting Information).

**TABLE 1:** Participant sociodemographic characteristics and mean childhood adiposity measures among MIREC study participants.

	*n* (%)	zBMI	*n* (%)	zWC	*n* (%)	%BF
		Mean (SD)		Mean (SD)		Mean (SD)
Total	234 (100)	0.21 (1.11)	233 (100)	0.23 (0.79)	220 (100)	18.92 (5.40)
Birthing parent characteristics						
Race and ethnicity						
Non‐white	19 (8)	0.17 (1.02)	19 (8)	0.14 (0.85)	17 (8)	19.53 (4.92)
White	215 (92)	0.22 (1.12)	214 (92)	0.24 (0.79)	203 (92)	18.87 (5.44)
Maternal age during pregnancy (years)						
< 30	72 (31)	0.20 (1.25)	72 (31)	0.24 (0.89)	68 (31)	18.71 (5.75)
30–34	91 (39)	0.26 (0.99)	90 (39)	0.25 (0.72)	89 (40)	18.84 (5.05)
35+	71 (30)	0.17 (1.10)	71 (30)	0.19 (0.79)	63 (29)	19.25 (5.53)
Education levels						
College, trade school diploma or less	71 (30)	0.20 (1.22)	71 (30)	0.20 (0.93)	69 (31)	19.74 (6.45)
Undergraduate degree	95 (41)	0.22 (1.13)	94 (40)	0.21 (0.77)	89 (40)	18.82 (5.03)
Graduate degree	68 (29)	0.21 (0.96)	68 (29)	0.28 (0.67)	62 (28)	18.14 (4.49)
Marital status						
Married	159 (68)	0.23 (1.05)	158 (68)	0.23 (0.78)	148 (67)	19.02 (5.33)
Not married	75 (32)	0.18 (1.23)	75 (32)	0.24 (0.83)	72 (33)	18.71 (5.56)
Smoking status during pregnancy						
Never	158 (68)	0.08 (1.03)	157 (67)	0.14 (0.80)	146 (66)	18.26 (5.24)
Ever	76 (32)	0.48 (1.21)	76 (33)	0.41 (0.75)	74 (34)	20.21 (5.51)
Country of birth						
Canada	205 (88)	0.24 (1.11)	204 (88)	0.25 (0.79)	196 (89)	18.99 (5.43)
Foreign	29 (12)	−0.02 (1.08)	29 (12)	0.12 (0.80)	24 (11)	18.34 (5.22)
Parity						
0	102 (44)	0.19 (1.20)	102 (44)	0.24 (0.87)	96 (44)	19.05 (5.82)
1	91 (39)	0.13 (1.03)	91 (39)	0.18 (0.73)	85 (39)	18.63 (5.26)
2+	41 (18)	0.45 (1.04)	40 (17)	0.29 (0.72)	39 (18)	19.22 (4.66)
Pre‐pregnancy BMI^a^ (kg/m^2^)						
< 25.0	140 (63)	−0.05 (1.00)	140 (63)	0.04 (0.70)	131 (62)	17.63 (4.48)
25.0–29.9	50 (23)	0.44 (0.05)	50 (23)	0.41 (0.80)	48 (23)	20.40 (5.27)
≥ 30.0	32 (14)	0.95 (1.39)	31 (14)	0.74 (0.96)	31 (15)	22.58 (7.27)
Child characteristics						
Sex assigned at birth						
Male	101 (43)	0.31 (1.15)	101 (43)	0.20 (0.75)	95 (43)	17.80 (4.50)
Female	133 (57)	0.14 (1.07)	132 (57)	0.25 (0.83)	125 (57)	19.77 (5.86)
Age at late childhood (years)						
Mean (SD)	8.9 (0.8)		8.9 (0.8)		8.9 (0.7)	
Physical activity (average minutes of moderate or vigorous activity/day)^a^						
Mean (SD)	61.9 (22.6)		62.1 (22.6)		62.4 (22.9)	

^a^ Missing covariates: pre‐pregnancy BMI (*n* = 13) and physical activity (*n* = 93).

We detected all metals in over 90% of study participants, with the exception of third trimester Hg (87%) (Table S1). We observed good reproducibility (ICC ≥ 0.40) between first and third trimester metals except for Mn (ICC = 0.28) and good reproducibility between early and late childhood Mn (ICC = 0.61) and Pb (ICC = 0.54) only. When examined across all time points, all metals showed low reproducibility (ICC < 0.40) except for Pb (ICC = 0.50) (Table S2). Furthermore, prenatal concentration of Hg was on average twice the measurement of childhood concentration (Figure S4).

### 3.2 Selection of Exposures and Interaction Effects

**FIGURE 1:**
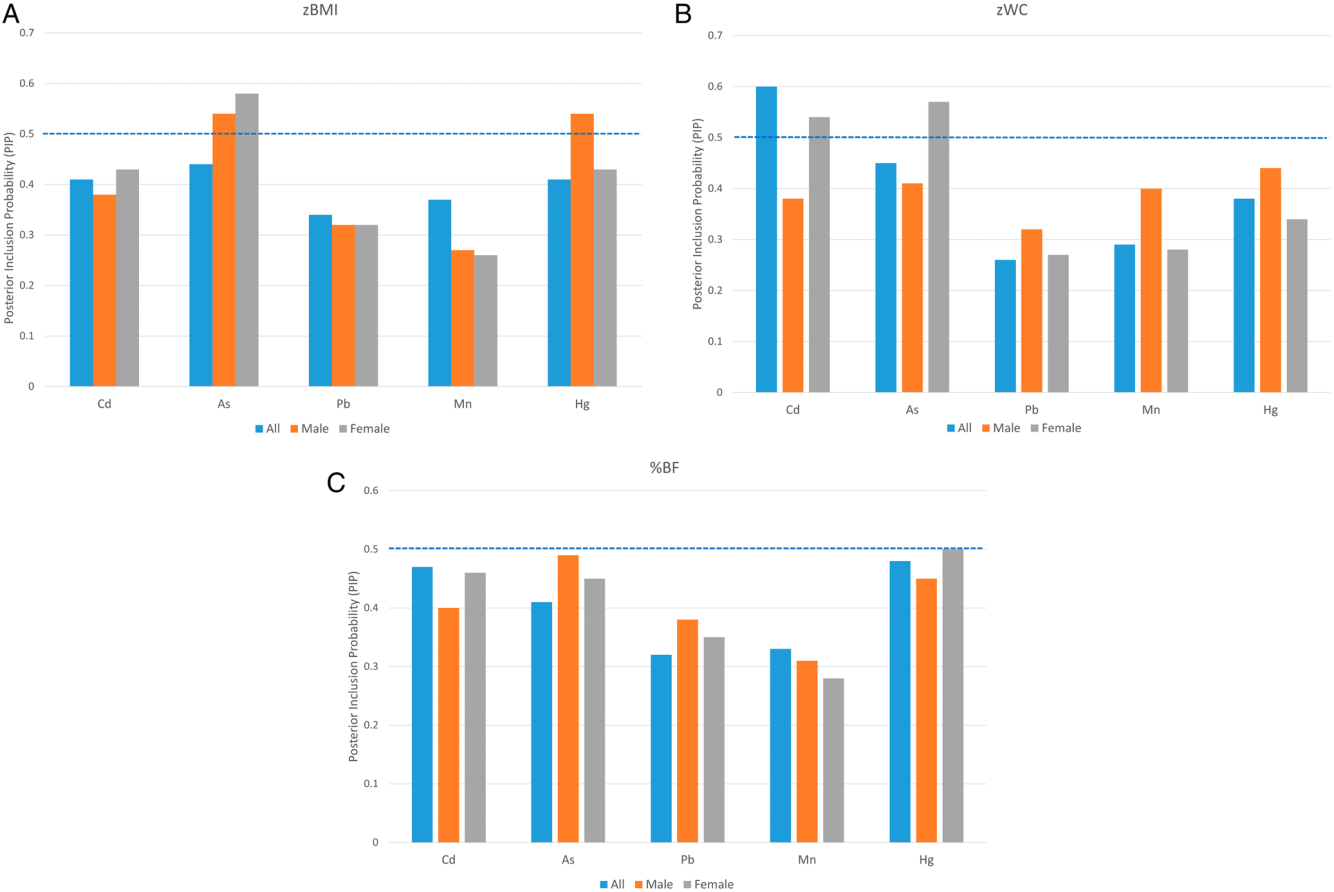
Posterior inclusion probabilities (PIPs) for adiposity models, using treed distributed lag mixture model (TDLMM) with threshold at 0.50. Blue bars represent the models with all children; orange bars represent sex‐specific models with male children; grey bars represent sex‐specific models with female children. zBMI and zWC models were adjusted for maternal race and ethnicity, birth country, education, age, pre‐pregnancy BMI, smoking, parity, marital status, clinic site and child's physical activity. %BF models additionally adjusted for child's age (all models) as well as sex assigned at birth for the full sample models. (A) zBMI. (B) zWC. (C) %BF.

In our main adjusted TDLMM models for zBMI, zWC and %BF including all children (blue bars in Figure 1), all PIPs were below the 0.50 threshold, with the exception of Cd in the zWC model. Our sex‐specific analyses yielded five exposures with PIPs above the 0.50 threshold for zBMI and zWC models. In the zBMI models, As was identified as important in female children (grey bars) and both As and Hg were identified as important in male children (orange bars). In the zWC models, both Cd and As had PIPs that exceeded the 0.50 threshold and were identified as important in female children. TDLMM did not select any exposures in the %BF models. It also did not identify any important pairwise interactions in any of the models.

### 3.3 Associations of Blood Metal Concentrations and Childhood Adiposity Measures

**FIGURE 2:**
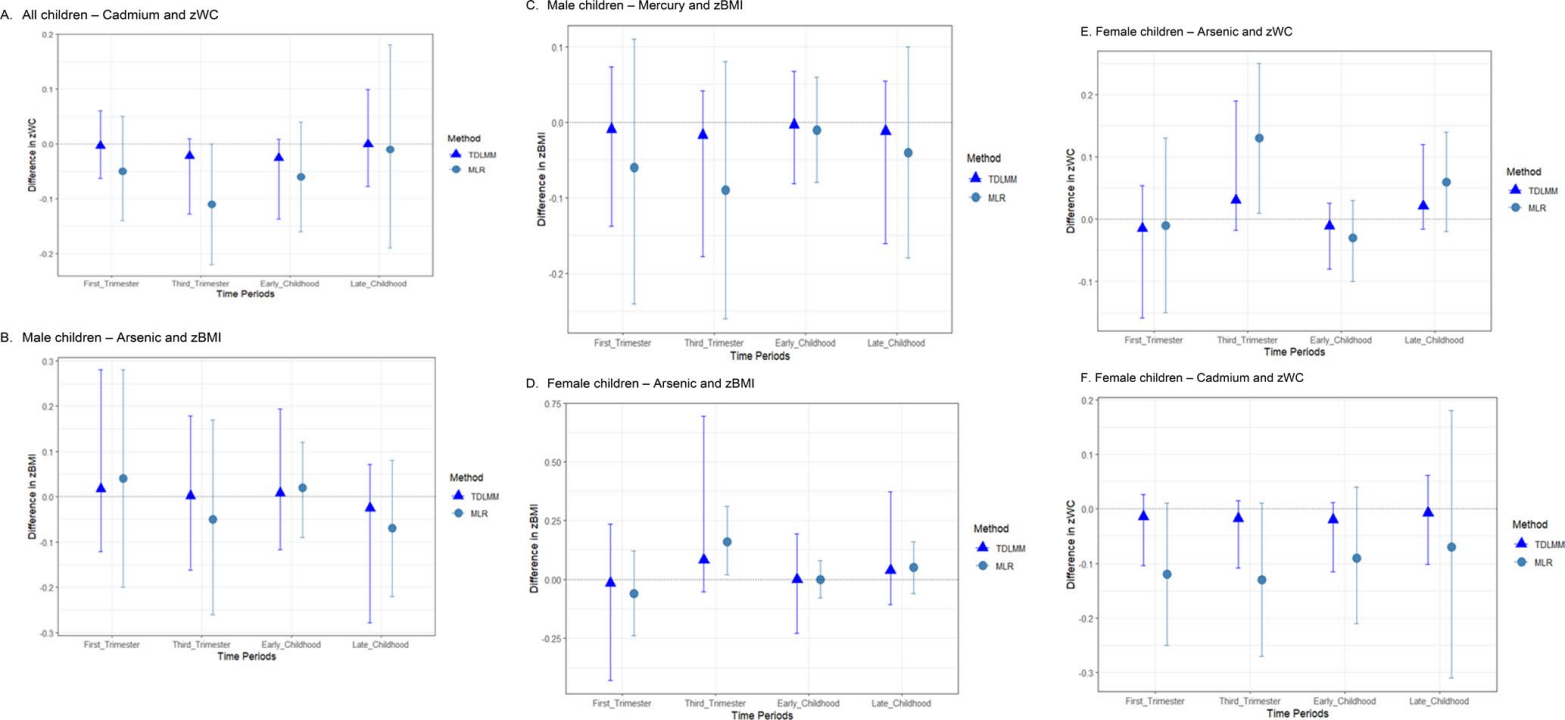
Adjusted effect estimates and plots showing the differences in adiposity measures and 95% CI associated with exposures to metals across various time periods, using TDLMM and multivariable linear regression. (A) Cd‐zWC association among all children. (B) As‐zBMI association among male children. (C) Hg‐zBMI association among male children. (D) As‐zBMI association among female children. (E) As‐zWC association among female children. (F) Cd‐zWC association among female children.

We estimated main effects in adjusted TDLMM and MLR models for the metal‐adiposity associations with PIPs > 0.50. Results were largely null with imprecise 95% credible intervals (CrI) in the TDLMM models; some MLR models were statistically significant (Figure 2, Table S3 and Figure S5). In the TDLMM adjusted main model that included all children, Cd showed a U‐shaped association with zWC over time; effect estimates were all close to the null and negative for measurements in the third trimester and early childhood with effect estimates −0.02 (95% CrI: −0.13, 0.01) and −0.03 (95% CrI: −0.14, 0.01), respectively. In the MLR models, the effect estimates were also in the same direction but with greater magnitude. Every two‐fold increase in third trimester and early childhood Cd concentrations was associated with a 0.11 (95% Confidence Interval (CI): −0.22, −0.00) and a 0.06 (95% CI: −0.16, 0.04) decrease in zWC, respectively, after adjusting for covariates (Figure 2A).

Among male children, the associations between both As and Hg and zBMI were null. The TDLMM effect estimates ranged from −0.03 (95% CrI: −0.28, 0.07) to 0.02 (95% CrI: −0.12, 0.28) for As and −0.02 (95% CrI: −0.18, 0.04) to −0.00 (95% CrI: −0.08, 0.07) for Hg. Our MLR models corroborated the null results (Figure 2B,C). Meanwhile, among female children, third trimester and late childhood (aged 7–9) As were positively associated with childhood zBMI and zWC (Figure 2D,E). For both outcomes, the magnitude of effect was strongest for the MLR models of third trimester measurements. Each two‐fold increase in third trimester As concentrations was associated with increases of 0.16 (95% CI: 0.02, 0.31) in zBMI and 0.13 (95% CI: 0.01, 0.25) in zWC, after adjusting for covariates (Figure 2D,E). For Cd exposure in female children, the adjusted MLR models showed negative associations in the first and the third trimesters (*p* < 0.1). Each doubling of Cd concentrations in the first and third trimester was associated with a 0.12 (95% CI: −0.25, 0.02) decrease and a 0.13 (95% CI: −0.27, 0.01) decrease in zWC, respectively (Figure 2F).

## 4 Discussion

Our investigation of the association between childhood adiposity measures and metal exposure at four time points from prenatal to late childhood (age 7–9) suggests that the third trimester may be a potential period of heightened susceptibility for As exposure, particularly for female children. We also observed some suggestion of inverse associations between third trimester Cd and childhood adiposity. However, for all other metals and time points, results were primarily null. Although the magnitude of associations we observed was small, the overlap in findings between the TDLMM and MLR models and measures of adiposity suggests a small but relatively robust association. This finding is consistent with the evidence from human and animal studies indicating that the origins of obesity can be traced back to the intrauterine environment [28, 29, 30, 31]. According to Barker's foetal programming hypothesis and the Developmental Origins of Health and Disease (DOHaD) theory, exposure to suboptimal prenatal environments such as in utero exposures to environmental stressors (e.g., environmental chemicals) can predispose the foetus to postnatal diseases [31]. Furthermore, these periods of heightened susceptibility may coincide with the timing of rapid cell differentiation [31].

During the third trimester, the foetus undergoes rapid adipogenesis [32]. This susceptible period is subject to environmental influences such as maternal nutrition and environmental chemical exposures [33]. As is a potential obesogen that can impair basic metabolic functions of adipocytes [34]. However, the underlying mechanisms of how prenatal exposure to As (especially during the third trimester) may increase childhood adiposity measures are not fully understood. Two mechanisms of how As affects adiposity have been proposed: increased fat content and epigenetic changes. As may act directly on adipose cells to cause adipocyte hyperplasia (an increase in the number of adipocytes) which increases total body fat mass [7, 35]. As may also interrupt adipocyte differentiation through epigenetic mechanisms, which in turn leads to decreased expression of genes such as peroxisome proliferator‐activated receptor γ, fatty acid‐binding protein and glucose transporter‐4 (*SLC2A4*) [36]. Furthermore, As may also affect adipokines such as leptin and adiponectin, which are cell‐signalling molecules associated with adipogenesis [36]. Lastly, prenatal exposure to As may disturb the differentiation and function of foetal mesenchymal stem cells (multipotent stem cells found in bone marrow) leading to impaired adipogenesis and changes in gene expressions involved in metabolic diseases [36]. Any resulting adipose tissue dysfunction is a substantial risk factor for childhood obesity [37].

Sex‐specific effects of As exposure have been reported; females are particularly vulnerable to potential As‐exposure related adverse birth outcomes [38, 39], congenital cardiac issues [40, 41] and neurodevelopmental disorders [42]. A number of these epidemiological studies have also reported adverse health effects (i.e., birth outcomes and congenital cardiac issues) associated with third trimester As exposure [38, 39, 40, 41]. However, the specific physiological mechanisms remain unclear.

Prior epidemiological studies examining the influence of prenatal As and metal mixtures on childhood adiposity measures are limited and the available research showed heterogeneous results [9, 10, 43, 44]. Agay‐Shay et al. [44] examined 11 s trimester maternal whole blood metals and metal mixture using Bayesian kernel machine regression and linear regression and found no associations with BMI, %BF and other cardiometabolic biomarkers among 411 Mexican children aged 4–6. Likewise, Agay‐Shay et al. [44], in their single pollutant and principal component analysis of multiple endocrine disrupting chemicals which included first and third trimester urinary Cd, As and Pb and cord blood Hg, also reported no association with BMI among 657 Spanish children with an average age of 7. In contrast, Smith et al. [10], who used quantile g‐computation to examine the associations between exposure to metal mixtures during the first trimester among 999 American children, reported that a non‐essential metal mixture consisting of As, Cd, Pb, Hg, caesium and barium was positively associated with BMI (*β* = 0.24; 95% CI: 0.07, 0.41) among children with a mean age of 8.

Studies that have examined concurrent metal exposure in children have also reported associations with adiposity measures [9, 45]. For example, Salcedo‐Bellido et al. [9] found that, in their case–control study (*n* = 92 controls, 51 cases), exposure to concurrent urinary metal mixture was associated with a 2‐fold increase in the risk of being overweight and having obesity in Spanish children aged 6–12. Their weighted quantile sum regression results indicated that Pb, Cd and As were the top three contributors to the adverse effect. In their single pollutant logistic regression analysis, children in the third tertile of As levels showed a 2‐fold higher odds of overweight and obesity compared to those in the first tertile. Furthermore, a recent single pollutant study also reported a 4‐fold increase in obesity risk when examining the associations between concurrent urinary As exposure and BMI but null associations with WC among 106 Iranian children and adolescents aged 6–18 [45]. Collectively, the studies provided evidence that non‐essential metals may affect adiposity measures and exposure timing may be key. This is reflected in our observations. However, differences in study design, sample size, participant characteristics, exposure assessment and statistical methods may have contributed to the magnitude differences and inconsistent results with different adiposity measures.

Besides As, Cd has been shown to affect adipose tissue and glucose metabolism [34, 46], increase oxidative stress [46] and act as an endocrine disruptor [47], all of which are potential biological mechanisms through which toxic metals could be related to higher adiposity measures. However, Cd's negative association with adiposity and obesity has also been reported previously, but the mechanism has not been elucidated. Similarly to our findings, Moynihan et al. [48] found that third trimester Cd exposure was negatively associated with adiposity measures among 185 female Mexican children aged 8–15. Shan [49], who examined concurrent metal exposure with obesity among American children aged 6 and 19, also reported negative associations between Cd levels and overweight and obesity. They, however, did not conduct sex‐specific analysis but reported an age‐related trend where higher Cd concentrations have greater effects on younger children compared to older children. Smith et al. [10], an aforementioned first trimester metal mixture study that showed a positive association with BMI among American children, had identified Cd as individually associated with a small negative association in female adolescents. They, however, found no association between Cd and adiposity measures in mid‐childhood (mean age (SD) = 8.0 (0.84)).

To our knowledge, this is the first study that examined the periods of heightened susceptibility to childhood obesity risk with exposure to multiple metals of concern. With four exposure time points, we used TDLMM, a distributed lag mixture method, and multiple linear regression to identify periods of heightened susceptibility and the most important contributors to our adiposity measures. Although our approach accounts for multi‐testing and the potential for chance findings given multiple metals, multiple time points and multiple adiposity measures, our findings should be interpreted with caution due to the following limitations. First, TDLMM is designed for mixtures of exposures observed at high temporal resolution, such as those of air pollution, but has also been shown to work well for a smaller number of time points [50]. TDLMM uses PIPs to identify the important metals but due to the small sample size of our analysis, the estimates are imprecise. As a result, we used both TDLMM and MLR results to identify any period of heightened susceptibility. Second, we applied MICE imputation to our missing chemical concentrations. Some exposure measurements were systematically missing (participants from certain clinic sites have unequal missingness due to clinic closure during the Covid pandemic) and we imputed the missing observations based on steps provided by Wijesuriya et al. [25] and van Buuren and Groothuis‐Oudshoorn [26] (Appendices B and C in Supporting Information). Analysis results were comparable after imputation (Appendix C Table B in Supporting Information). Third, our sample had mostly individuals with healthy BMI, WC and %BF. This narrow variability may have limited our ability to observe effects. Furthermore, we did not observe an association with %BF. This is likely reflective of the differences in weight distribution rather than fat percentage. Fourth, due to data availability, we only assessed total As and did not distinguish inorganic As from the generally less toxic organic As forms. Also, we were unable to assess the effects of other metals and control for dietary factors. However, we were able to adjust for physical activity levels using a gold‐standard field‐based measure. Lastly, the MIREC, MIREC‐CD Plus and MIREC‐ENDO participants are predominantly white and of higher socioeconomic status [12]. As a result, the generalisability of our results may be limited. Considering these limitations, our findings warrant future studies that include larger populations with heterogeneous sociodemographic and metabolic characteristics and have detailed exposure data, including both total and speciated As.

## 5 Conclusion

The aetiology of obesity is complex and not fully elucidated. Our findings suggest that the third trimester in pregnancy may be a period of heightened susceptibility to the obesogenic effects of As exposure, particularly among female children aged 7–9. Future research is needed to replicate these findings and examine why females may be more vulnerable than males to the negative impact of prenatal As exposure, especially during late pregnancy. Better understanding the role of early exposure to contaminants on obesity risk will allow for environmental health prevention opportunities during pregnancy and early life, which is critical to children's health.

## Author Contributions

J.M.Y.H.: conceptualisation, methodology, formal analysis and writing. M.M.B., J.M.B., K.M.M., M.R.P., L.B. and C.P.: review and editing. J.A.‐M.: supervision, funding acquisition, review and editing. All authors were involved in editing the paper and had final approval of the submitted and published versions.

## Ethics Statement

Research Ethics Board approval was obtained at Health Canada/Public Health Agency of Canada and at the Study Coordinating Centre (Sainte Justine University Hospital Centre (Montreal, QC, Canada)) and at all study sites; all study participants provided informed consent prior to participation.

## Conflicts of Interest

The authors declare no conflicts of interest.

## Supporting information


**Data S1:** ijpo70057‐sup‐0001‐DataS1.pdf.

## Data Availability

Due to data privacy issues, we are not able to make these data publicly available. Individuals may apply to access the data through the MIREC Biobank (www.mirec‐canada.ca/en/research).
