# Supplementary material for: Exposure to Metal Mixtures and Childhood Adiposity: An Examination of Periods of Heightened Susceptibility Between Gestation and Late Childhood
Source: Pediatr Obes. 2025 Sep 14;20(12):e70057. doi: 10.1111/ijpo.70057 (PMC12590100; doi:10.1111/ijpo.70057)
Supplement: Supplementary file 1 — Data S1: ijpo70057‐sup‐0001‐DataS1.pdf. [file IJPO-20-e70057-s001.pdf]

Manuscript Title: Exposure to metal mixtures and childhood adiposity: An examination of periods of heightened susceptibility between gestation and late childhood

Authors:
Janice M.Y. Hu[a], Michael M. Borghese[a], Mandy Fisher[a], Joseph M. Braun[b], Katherine M. Morrison[c], Mark R. Palmert[d], Linda Booij[e,f], Constadina Panagiotopoulos[g], Jillian Ashley-Martin[a]

Affiliations:
[a] Environmental Health Science and Research Bureau, Healthy Environments and Consumer Safety Branch, Health Canada, Ottawa, ON, Canada
[b] Department of Epidemiology, Brown University, Providence, RI, USA
[c] Department of Pediatrics, McMaster University, Hamilton, ON, Canada
[d] Division of Endocrinology, Hospital for Sick Children and Departments of Pediatrics and Physiology, University of Toronto, Toronto, ON, Canada
[e] Department of Psychiatry and Douglas Mental health University Institute, McGill University, Montreal, QC, Canada
[f] CHU Sainte-Justine Azrieli Research Center, Montreal, QC, Canada
[g] Department of Pediatrics, University of British Columbia, Vancouver, BC, Canada

Corresponding author:
Janice Hu, Environmental Health Science and Research Bureau, Health Canada, 251 Sir Frederick Banting Way, Ottawa, ON, Canada, K1A 0K9; Janice.Hu@hc-sc.gc.ca

Supplemental Tables, Figures and Appendices:

Figure S1. Study sample flowchart 1

Figure S2. Project overview 2

Figure S3. Directed Acyclic Graph for the relation among metal concentrations at various time points, childhood adiposity measures, and various demographic characteristics 3

Table S1. Descriptive statistics of whole blood metal concentrations during pregnancy and childhood 4-5

Figure S4. Whole blood metal geometric mean concentrations (µg/L) over four time points

.... 6-7

Table S2. Intra-class correlations (ICC) of blood metal concentrations 8

Table S3. Adjusted differences in adiposity measures and 95%CI associated per doubling in metal concentrations across various time periods, using TDLMM and multivariable linear regression 9-10

Figure S5. Adjusted effect estimate plots showing the differences in adiposity measures and 95%CI associated with exposures to metals across various time periods, using multivariable linear regression 11-13

Appendix A. Laboratory analysis 14

Appendix B. MICE imputation... 15

Appendix C. Complete case analysis results 16-17

Supplemental Tables, Figures and Appendices

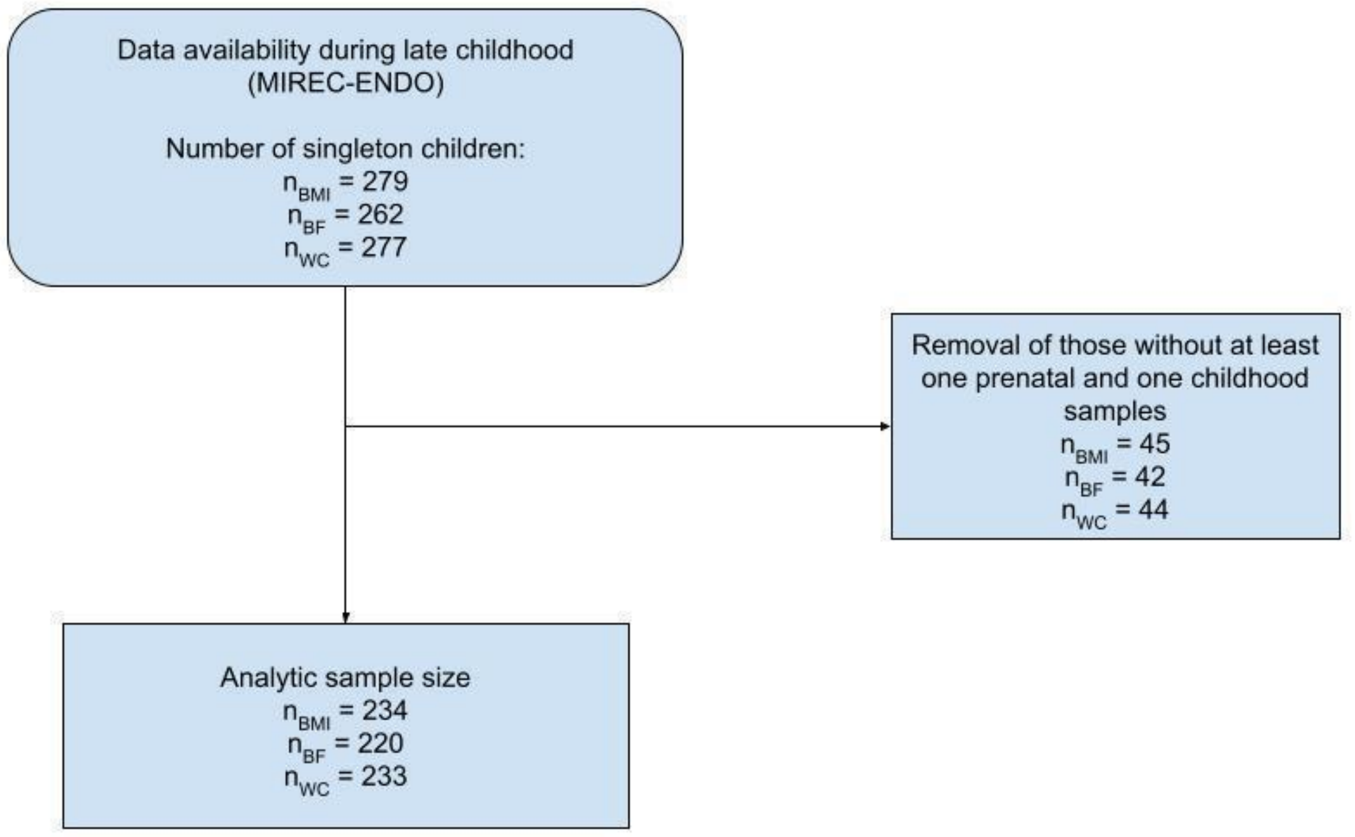

Figure S1. Study sample flow chart.

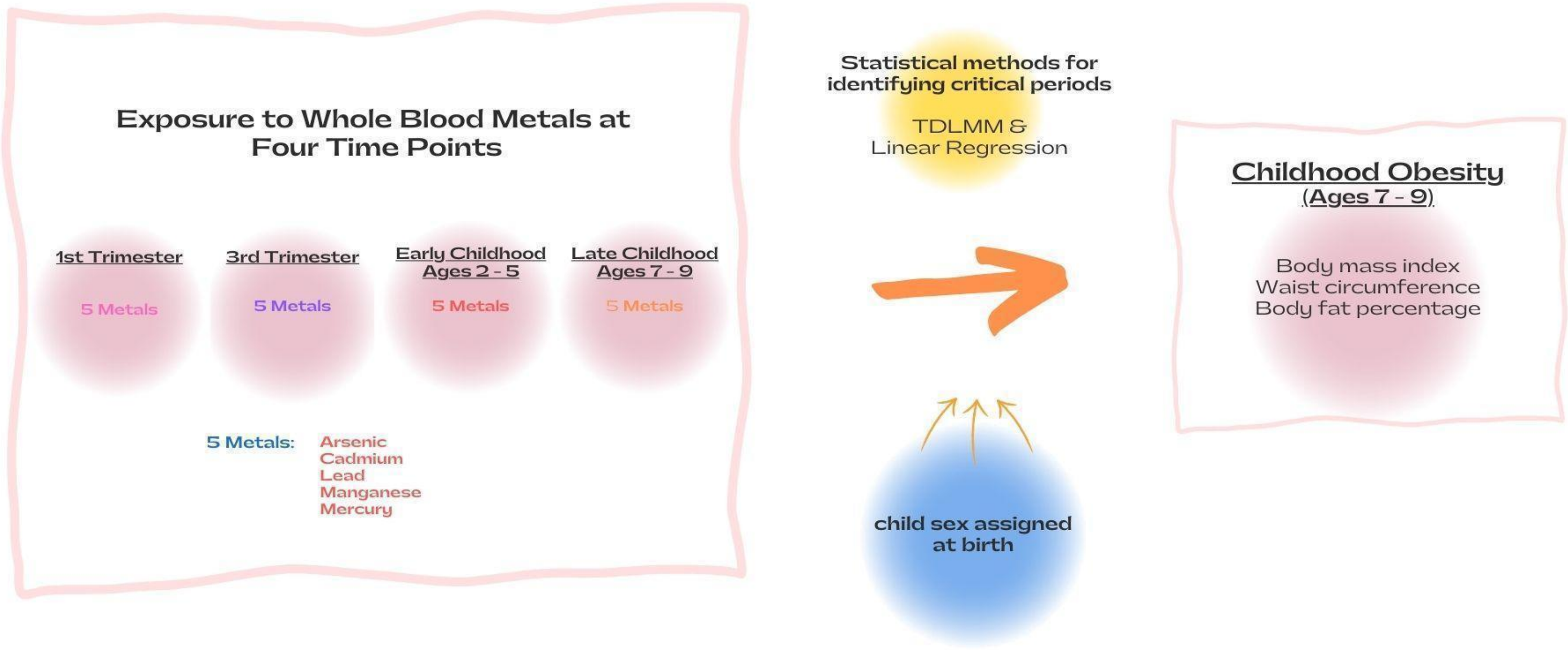

Figure S2. Project overview.

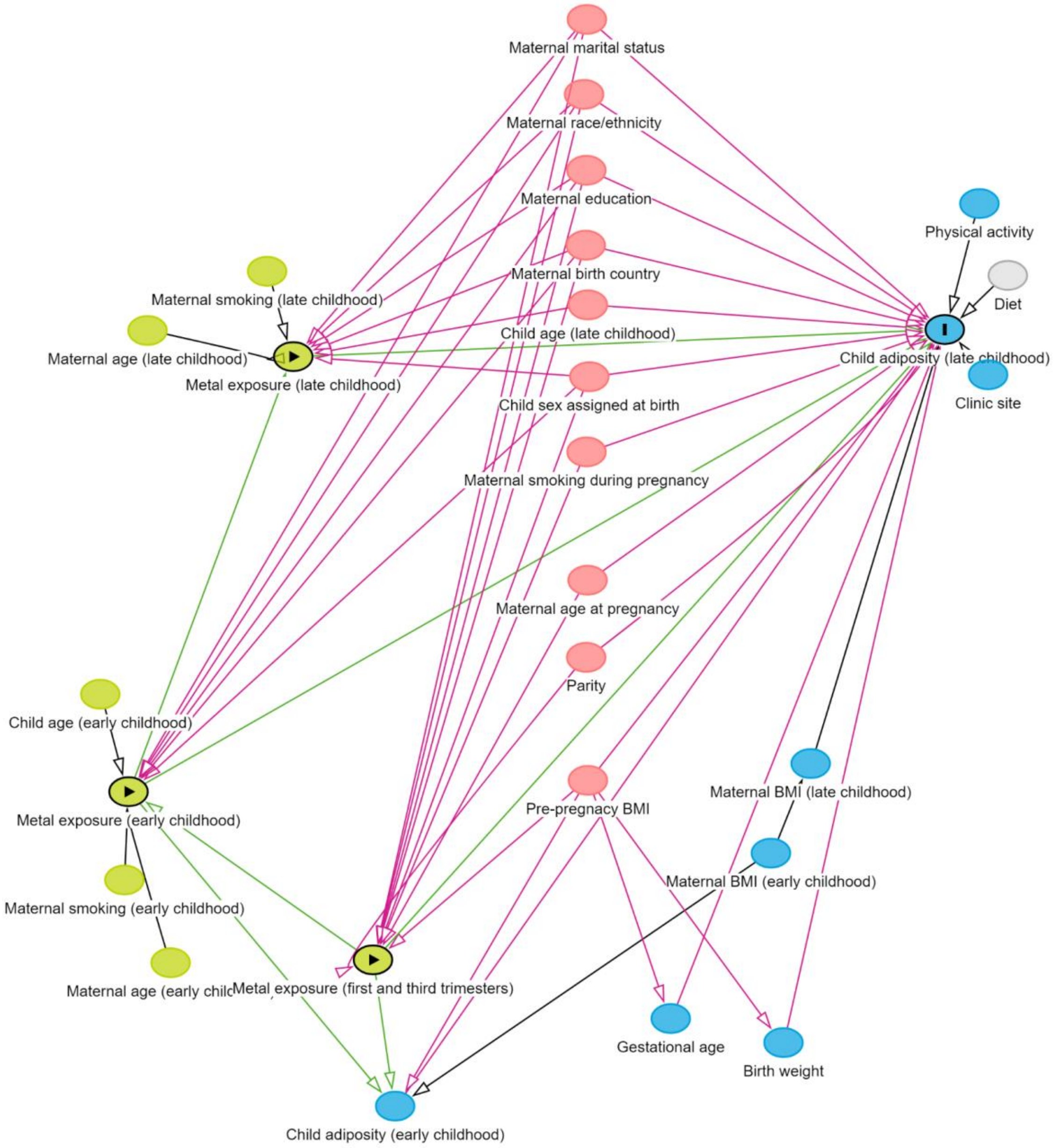

Figure S3. Directed Acyclic Graph for the relation among metal concentrations at various time points, childhood adiposity measures, and various demographic characteristics.

Table S1. Descriptive statistics of whole blood metal concentrations during pregnancy and childhood.								
	LOD*	%>LOD	GM*	Min	25th	50th	75th	95th
**First trimester whole blood metal concentrations (µg/L)**								
Arsenic (As)	0.22	95	0.8	<LOD	0.5	0.8	1.2	2.3
Cadmium (Cd)	0.04	98	0.2	<LOD	0.1	0.2	0.3	0.8
Mercury (Hg)	0.12	91	0.6	<LOD	0.3	0.7	1.2	2.7
Lead (Pb)**	1.04	100	6.3	1.6	4.6	6.1	8.5	13.4
Manganese (Mn)	0.55	100	8.7	2.6	7.1	8.8	10.4	13.7
**Third trimester whole blood metal concentrations (µg/L)**								
Arsenic (As)	0.22	91	0.7	<LOD	0.4	0.7	1.1	2.7
Cadmium (Cd)	0.04	99	0.2	<LOD	0.2	0.2	0.3	0.6
Mercury (Hg)	0.12	87	0.5	<LOD	0.2	0.5	0.9	1.3
Lead (Pb)**	1.04	100	6.0	1.6	4.1	5.8	8.5	13.4
Manganese (Mn)	0.55	100	12.0	3.3	9.9	12.1	14.3	19.6
**Early childhood (aged 2-5) whole blood metal concentrations*** (µg/L)**								
Arsenic (As)	0.22	100	0.4	<LOD	0.2	0.5	0.9	3.0
Cadmium (Cd)	0.04	100	0.1	<LOD	0.1	0.1	0.1	0.2
Mercury (Hg)	0.12	100	0.1	<LOD	<LOD	0.2	0.6	2.1

Lead (Pb)**	1.04	100	7.3	2.5	4.7	6.7	10.8	21.5
Manganese (Mn)	0.41	100	10.4	4.2	9.0	10.4	12.5	15.8
**Late childhood (aged 7-9) whole blood metal concentrations*** (µg/L)**								
Arsenic (As)	0.36	100	0.4	<LOD	<LOD	0.4	0.7	2.3
Cadmium (Cd)	0.06	100	0.1	<LOD	0.1	0.1	0.1	0.2
Mercury (Hg)	0.28	100	0.2	<LOD	<LOD	0.3	0.6	2.1
Lead (Pb)**	1.39	100	5.3	1.6	3.7	5.2	7.0	12.4
Manganese (Mn)	1.32	100	11.1	5.6	9.3	11.0	12.8	16.7

* LOD – limit of detection, GM – geometric mean
** Blood Pb concentrations are typically reported in µg/dL, we report all blood metal concentrations in µg/L for consistency across measures, in alignment with calls to standardize reporting values for blood Pb concentrations [61].
*** Machine reading values were used

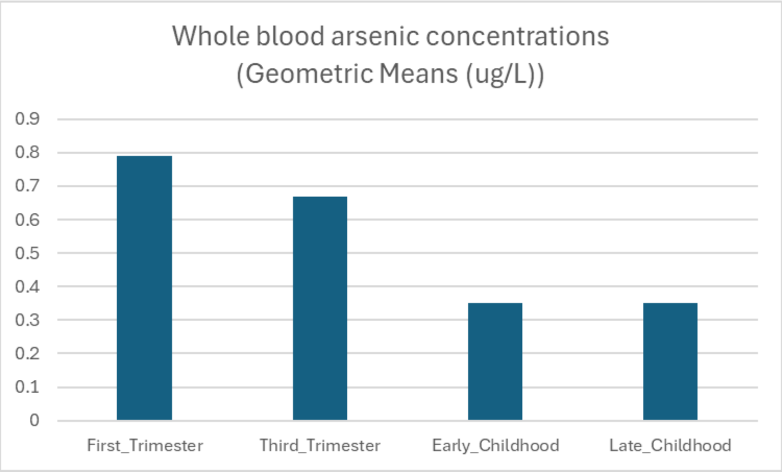
Whole blood arsenic concentrations
(Geometric Means (ug/L))
0.9
0.8
0.7
0.6
0.5
0.4
0.3
0.2
0.1
0
First_Trimester
Third_Trimester
Early_Childhood
Late_Childhood

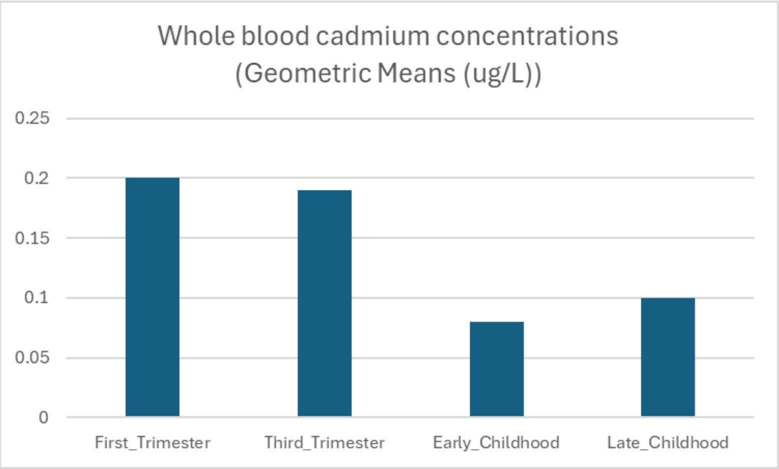
Whole blood cadmium concentrations
(Geometric Means (ug/L))
0.25
0.2
0.15
0.1
0.05
0
First_Trimester
Third_Trimester
Early_Childhood
Late_Childhood

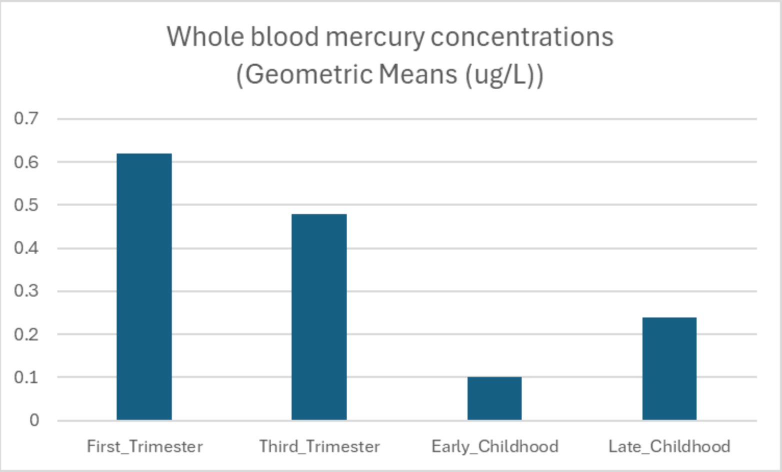
Whole blood mercury concentrations
(Geometric Means (ug/L))
0.7
0.6
0.5
0.4
0.3
0.2
0.1
0
First_Trimester
Third_Trimester
Early_Childhood
Late_Childhood

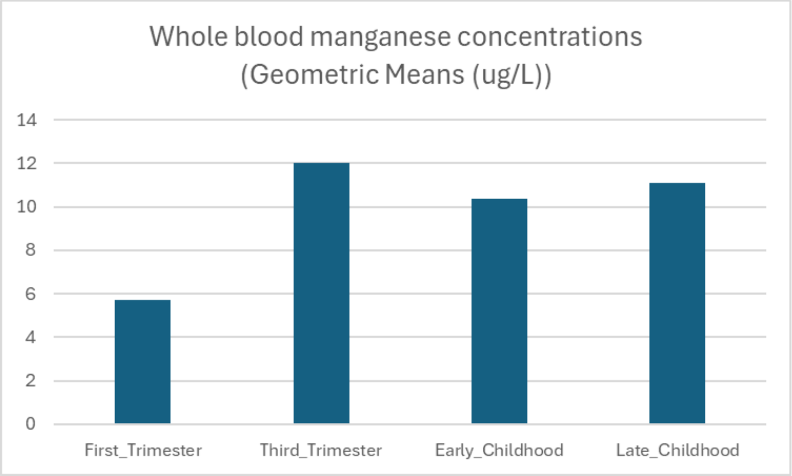
Whole blood manganese concentrations
(Geometric Means (ug/L))
14
12
10
8
6
4
2
0
First_Trimester
Third_Trimester
Early_Childhood
Late_Childhood

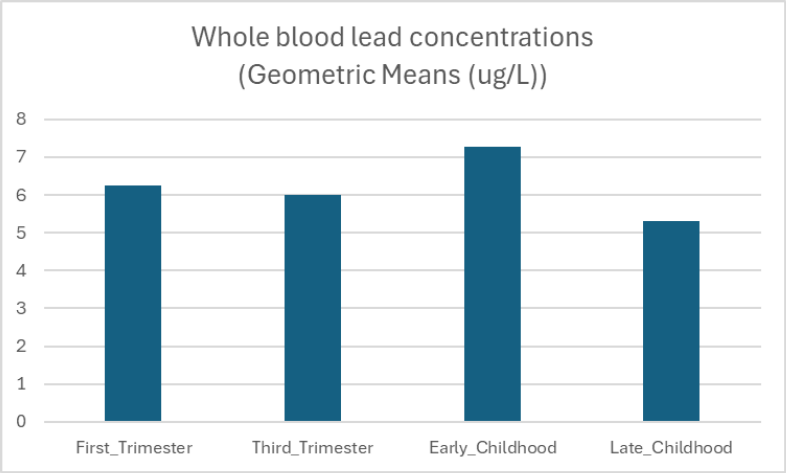

Figure S4. Whole blood metal geometric mean concentrations (µg/L) over four time points.

Table S2. Intra-class correlations (ICC) of blood metal concentrations. Measurements were taken at the first trimester, third trimester, early childhood (2-5 years old) and late childhood (7 to 9 years old).

Metals	ICC (First and third trimester)	ICC (Early and late childhood)	ICC (All time points)
Arsenic (As)	0.46	0.29	0.12
Cadmium (Cd)	0.68	0.19	0.08
Mercury (Hg)	0.73	0.20	0.16
Manganese (Mn)	0.28	0.61	0.34
Lead (Pb)	0.76	0.54	0.50

Table S3. Adjusted differences in adiposity measures and 95%CI associated per doubling in metal concentrations across various time periods, using TDLMM and multivariable linear regression.

	First trimester	Third trimester	Early childhood	Late childhood
A. All children – Cadmium and zWC				
TDLMM β (95% CrI)	-0.00 (-0.06, 0.06)	-0.02 (-0.13, 0.01)	-0.03 (-0.14, 0.01)	-0.00 (-0.08, 0.10)
MLR β (95% CI)	-0.05 (-0.14, 0.05)	-0.11 (-0.22, -0.00)	-0.06 (-0.16, 0.04)	-0.01 (-0.19, 0.18)
B. Male children – Arsenic and zBMI				
TDLMM β (95% CrI)	0.02 (-0.12, 0.28)	0.00 (-0.16, 0.18)	0.01 (-0.12, 0.19)	-0.03 (-0.28, 0.07)
MLR β (95% CI)	0.04 (-0.20, 0.28)	-0.05 (-0.26, 0.17)	0.02 (-0.09, 0.12)	-0.07 (-0.22, 0.08)
C. Male children – Mercury and zBMI				
TDLMM β (95% CrI)	-0.01 (-0.14, 0.07)	-0.02 (-0.18, 0.04)	-0.00 (-0.08, 0.07)	-0.01 (-0.16, 0.05)
MLR β (95% CI)	-0.06 (-0.24, 0.11)	-0.09 (-0.26, 0.08)	-0.01 (-0.08, 0.06)	-0.04 (-0.18, 0.10)
D. Female children – Arsenic and zBMI				
TDLMM β (95% CrI)	-0.02 (-0.43, 0.23)	0.08 (-0.05, 0.70)	-0.00 (-0.23, 0.19)	0.04 (-0.11, 0.37)
MLR β (95% CI)	-0.06 (-0.24, 0.12)	0.16 (0.02, 0.31)	0.00 (-0.08, 0.08)	0.05 (-0.06, 0.16)

E. Female children – Arsenic and zWC				
TDLMM<br>β (95% CrI)	-0.01<br>(-0.16, 0.05)	0.03<br>(-0.02, 0.19)	-0.01<br>(-0.08, 0.03)	0.02<br>(-0.02, 0.12)
MLR<br>β (95% CI)	-0.01<br>(-0.15, 0.13)	0.13<br>(0.01, 0.25)	-0.03<br>(-0.10, 0.03)	0.06<br>(-0.02, 0.14)
F. Female children – Cadmium and zWC				
TDLMM<br>β (95% CrI)	-0.01<br>(-0.10, 0.03)	-0.02<br>(-0.11, 0.02)	-0.02<br>(-0.12, 0.01)	-0.01<br>(-0.10, 0.06)
MLR<br>β (95% CI)	-0.12<br>(-0.25, 0.01)	-0.13<br>(-0.27, 0.01)	-0.09<br>(-0.21, 0.04)	-0.07<br>(-0.31, 0.18)
Models shown were selected based on TDLMM PIPs.				

A. zBMI

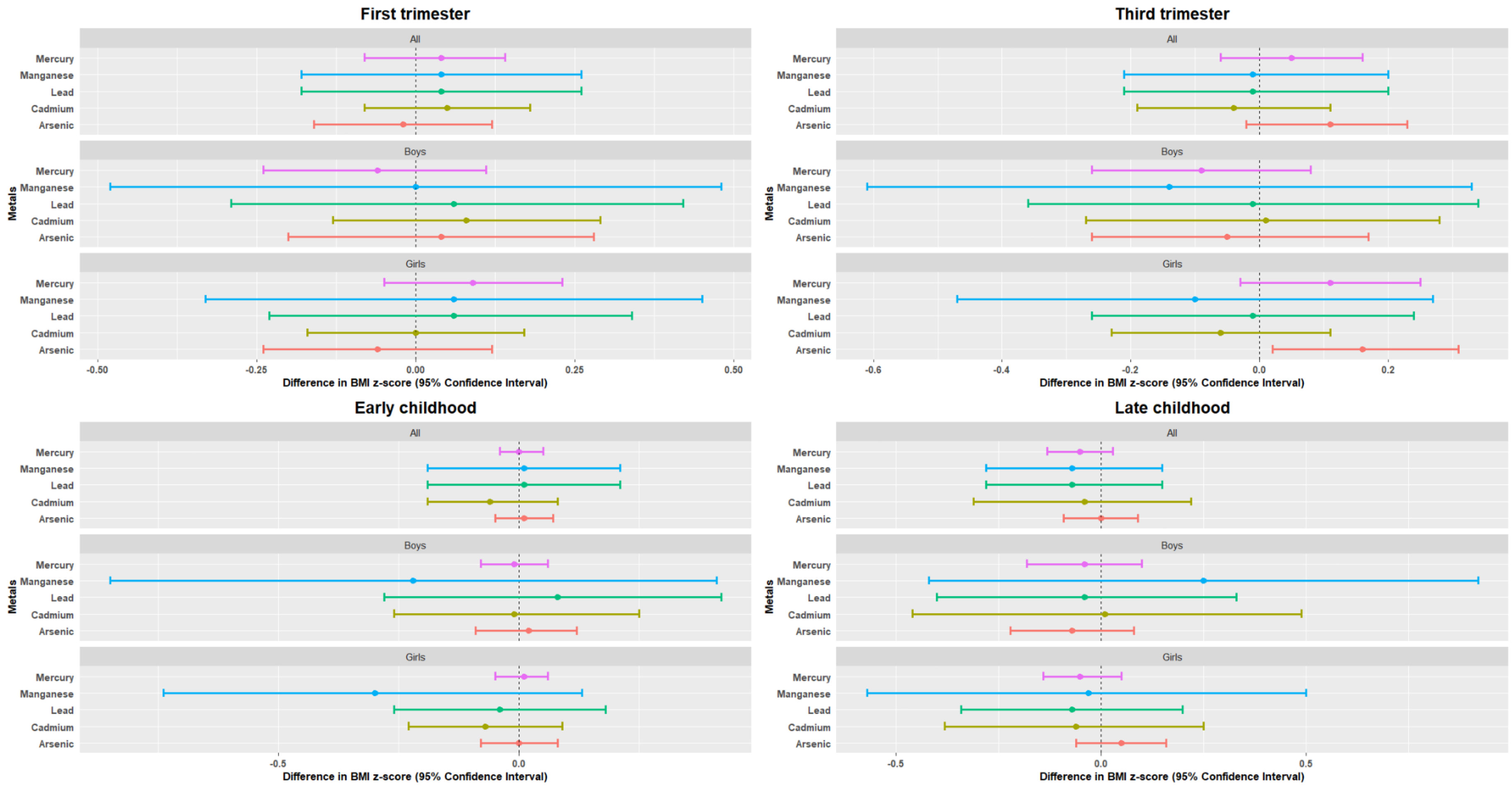
First trimester
Third trimester
Early childhood
Late childhood
All
Boys
Girls
Mercury
Manganese
Lead
Cadmium
Arsenic
Metals
-0.50
-0.25
0.00
0.25
0.50
-0.6
-0.4
-0.2
0.0
0.2
-0.5
0.0
0.5
Difference in BMI z-score (95% Confidence Interval)

B. zWC

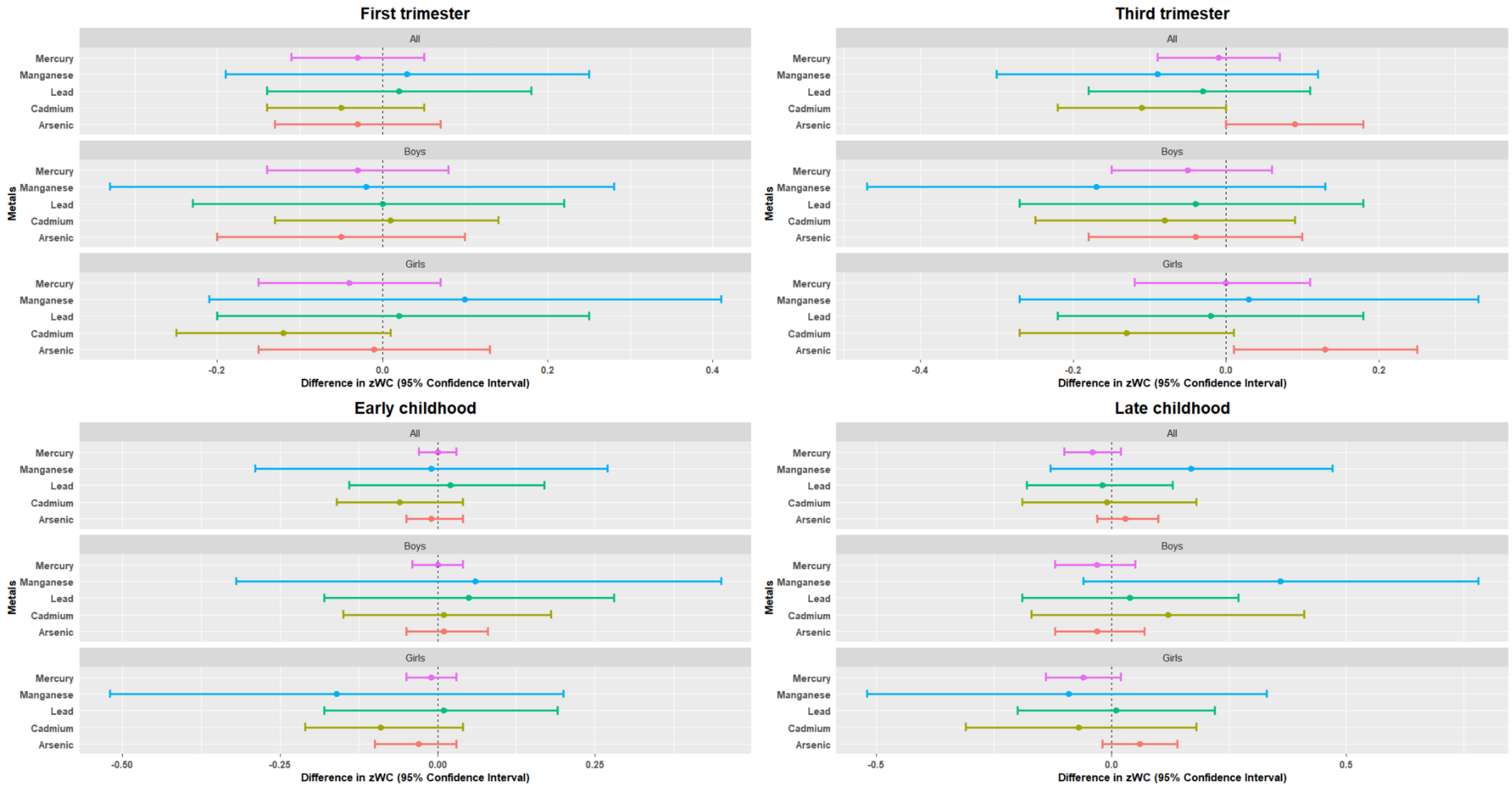

First trimester
Third trimester
Early childhood
Late childhood
All
Boys
Girls
Mercury
Manganese
Lead
Cadmium
Arsenic
Metals
Difference in zWC (95% Confidence Interval)

C. BF%

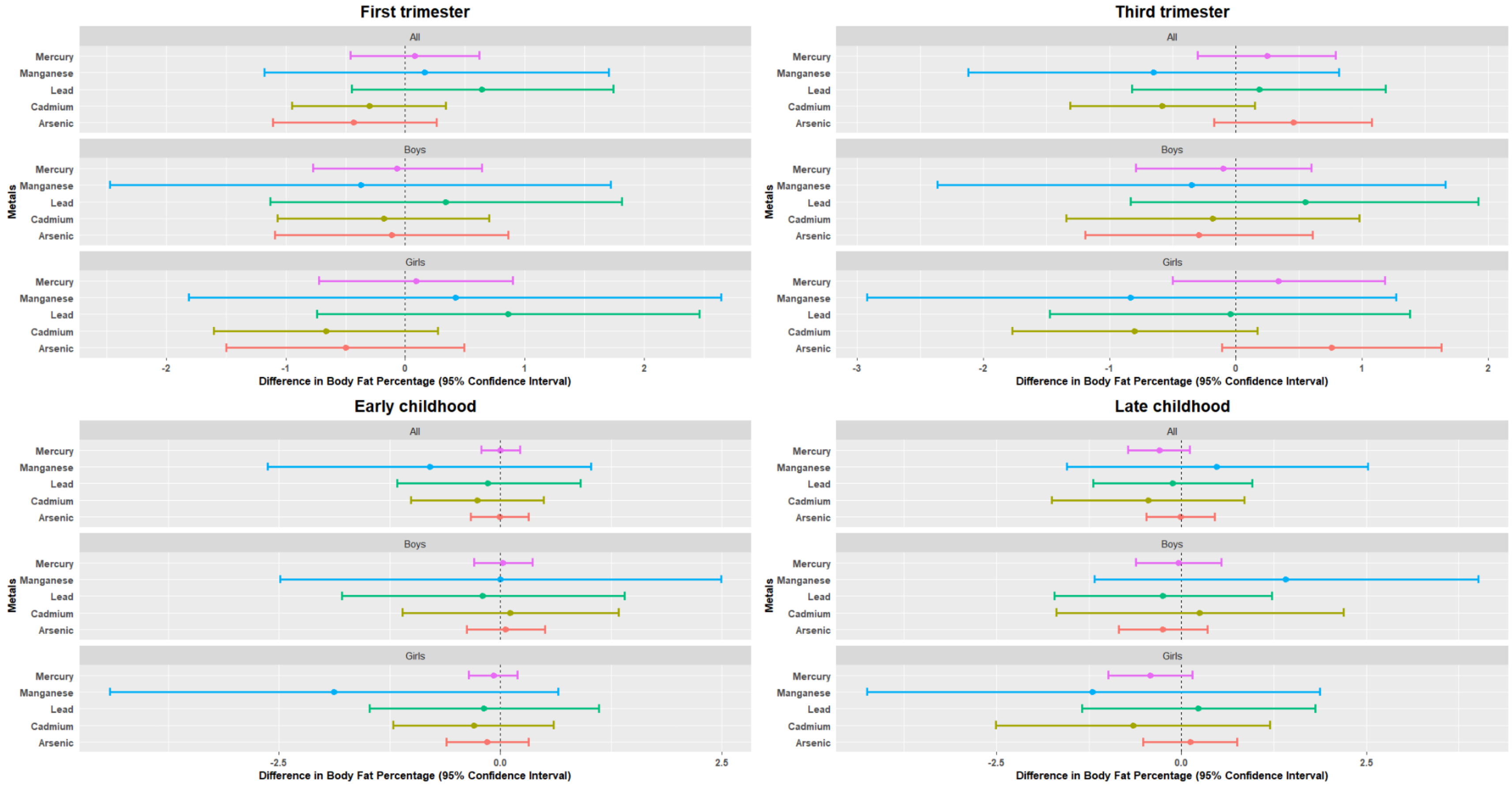

Figure S5. Adjusted effect estimate plots showing the differences in adiposity measures and 95%CI associated with exposures to metals across various time periods, using multivariable linear regression. **A.** zBMI. **B.** zWC. **C. %**BF.

Appendix A. Laboratory analysis

The Toxicology Laboratory of the Institut national de santé publique du Québec performed the laboratory analysis for all time points. The laboratory analysis methods for samples collected during the first trimester, third trimester and early childhood were previously described [1,2]. The analytical method for metals in children's whole blood samples collected during late childhood is described below.

Concentrations of metals analyzed were measured in children's whole blood. The samples were diluted in a basic solution containing ethoxylate octylphenol and ammonium hydroxide, then analyzed by PerkinElmer NexION 300S inductively-coupled plasma-mass spectrometry system (ICP-MS). Calibration is performed using matrix-matched standards.

The limits of detection (LOD) for the metals were determined by using a signal-to-noise ratio of 3:1 obtained from 10 measurements of a representative sample. Concentrations were reported in units of nanomoles per litre (nmol/L) (except for lead, which was reported in micromoles per litre). The LODs varied from 0.49 to 24 nmol/L. Internal quality control was ensured by analyzing three reference materials from the Québec Multielement External Quality Assessment Scheme (QMEQAS).

[1] Arbuckle TE, Liang CL, Morisset AS, Fisher M, Weiler H, Cirtiu CM, et al. Maternal and fetal exposure to cadmium, lead, manganese and mercury: The MIREC study. *Chemosphere*. 2016;163:270-282.

[2] Ashley-Martin J, Dodds L, Arbuckle TE, Lanphear B, Muckle G, Bouchard MF, et al. Blood metal levels and early childhood anthropometric measures in a cohort of Canadian children. *Environ Res*. 2019;179(Pt A):108736.

Appendix B. MICE imputation

During Covid, some clinics were closed, which resulted in unequal missingness in exposure measurements. We imputed the missing exposure and covariate data for participants with at least one prenatal and one postnatal observation. We followed the single level fully conditional specification with dummy indicators for higher level clusters with repeated measures imputed in wide format method in Wijesuriya et al. [1]. It is a multiple imputation by chained equations (MICE) method [2] that allows the correlation among participants belonging to higher-level cluster (i.e., the clinics) to be modelled through dummy indicators while the correlation among the repeated measures (i.e., four time points) is modelled by including the repeated measures as distinct variables in the imputation model. In other words, the imputation method allows imputation of incomplete repeated measure at one time point by including repeated measures at all the other time points as predictors, thereby preserving the correlation of the repeated measures. We imputed 20 datasets and included all covariates, outcome variable, and exposure measurement at all four time points in the imputation models.

[1] Wijesuriya R, Moreno-Betancur M, Carlin JB, Lee KJ. Evaluation of approaches for multiple imputation of three-level data. *BMC Med Res Methodol*. 2020;20(1):207.

[2] van Buuren S, Groothuis-Oudshoorn K. mice: Multivariate Imputation by Chained Equations in R. *J Stat Softw*. 2011;45:1-67.

Appendix C. Complete case analysis results

The table below shows the descriptive statistics of the original dataset with 279, 277 and 262 children and their birthing parents for zBMI, zWC and %BF analysis, respectively. Note that the sample size decreased after imputation of the covariates and the chemical concentrations because we excluded participants without at least one prenatal and one postnatal chemical concentration measurements. For example, for zBMI, we excluded 42 participants without postnatal and 3 without prenatal observations (Figure S1).

Both datasets had comparable participant characteristics (Table 1 and Table A below). The mean (SD) child age when the adiposity measures were obtained was the same at 8.9 (0.8). The majority of children were female (56% original vs 57% imputed) and the children's were physically active, on average, for over 60 minutes per day. The majority of parents were white (90% original vs 92% imputed), born in Canada (87% original vs 88% imputed), married (70% original vs 68% imputed), over 30 years of age (71% original vs 69% imputed), with at least an undergraduate degree or higher (71% original vs 70% imputed), never smoked (67% original vs 68% imputed), and had a pre-pregnancy BMI < 25.0 kg/m^2 (63% for both datasets). Furthermore, 46% in the original dataset (vs. 44% imputed) were nulliparous. Characteristics of pregnant individuals with the adiposity measures were the same except that males instead of females tended to have higher zWC and that non-white individuals instead of white individuals tended to have higher zBMI.

Table A. Participant sociodemographic characteristics and mean childhood adiposity measures among MIREC study participants using original (unimputed) dataset..

	n (%)	zBMI Mean (SD)	n (%)	zWC Mean (SD)	n (%)	%BF Mean (SD)
Total	279 (100)	0.22 (1.11)	277 (100)	0.23 (0.79)	262 (100)	19.94 (5.66)
**Birthing Parent Characteristics**						
Race and ethnicity						
Non-white	27 (10)	0.29 (1.15)	27 (10)	0.22 (0.87)	24 (9)	20.02 (5.18)
White	252 (90)	0.21 (1.10)	250 (90)	0.23 (0.79)	238 (91)	18.83 (5.71)
Maternal age during pregnancy (years)						
<30	81 (29)	0.17 (1.22)	80 (29)	0.21 (0.88)	76 (29)	18.44 (5.66)
30-34	101 (36)	0.28 (1.02)	100 (36)	0.27 (0.72)	99 (38)	18.92 (5.45)

35+	97 (35)	0.19 (1.10)	97 (35)	0.22 (0.81)	87 (33)	19.42 (5.92)
Education levels						
College, trade school diploma or less	80 (29)	0.17 (1.20)	80 (29)	0.17 (0.91)	77 (29)	19.47 (6.34)
Undergraduate degree	120 (43)	0.26 (1.14)	118 (43)	0.23 (0.78)	111 (42)	19.17 (5.78)
Graduate degree	79 (28)	0.21 (0.97)	79 (29)	0.30 (0.68)	74 (28)	18.06 (4.60)
Marital status						
Married	196 (70)	0.24 (1.04)	198 (70)	0.24 (0.77)	181 (69)	19.04 (5.54)
Not married	83 (30)	0.16 (1.25)	84 (30)	0.21 (0.84)	81 (31)	18.73 (5.96)
Smoking status during pregnancy						
Never	187 (67)	0.07 (1.03)	185 (67)	0.13 (0.80)	173 (66)	18.16 (5.32)
Ever	92 (33)	0.52 (1.20)	92 (33)	0.44 (0.75)	89 (34)	20.46 (6.02)
Country of birth						
Canada	243 (87)	0.25 (1.11)	240 (87)	0.25 (0.80)	231 (88)	19.03 (5.72)
Foreign	36 (13)	0.04 (1.08)	37 (13)	0.14 (0.77)	31 (12)	18.30 (5.27)
Parity						
0	128 (46)	0.18 (1.15)	127 (46)	0.23 (0.84)	121 (46)	18.82 (6.02)
1	107 (38)	0.17 (1.06)	107 (39)	0.22 (0.76)	99 (38)	18.85 (5.37)
2+	44 (16)	0.45 (1.10)	43 (16)	0.27 (0.76)	42 (16)	19.52 (5.36)
Pre-pregnancy BMI* (kg/m^2)						
<25.0	167 (63)	0.02 (1.02)	167 (64)	0.10 (0.72)	156 (63)	17.99 (5.06)
25.0 - 29.9	56 (21)	0.37 (0.95)	39 (15)	0.32 (0.82)	53 (21)	19.96 (5.30)
≥30.0	41 (16)	0.77(1.38)	56 (21)	0.66 (0.92)	40 (16)	21.48 (7.08)
**Child Characteristics**						
Sex assigned at birth						
Male	122 (44)	0.38 (1.15)	122 (44)	0.25 (0.74)	115 (44)	18.16 (5.25)
Female	157 (56)	0.09 (1.06)	155 (56)	0.22 (0.84)	147 (56)	19.56 (5.91)
Age at late childhood (years)						
Mean (SD)	8.9 (0.8)		8.9 (0.8)		8.9 (0.8)	
Physical Activity (average minutes of moderate or vigorous activity/day)*						
Mean (SD)	62.0 (23.1)		62.0 (23.2)		62.4 (22.9)	

*Missing covariates: pre-pregnancy BMI (n = 13) and physical activity (n = 103)

Furthermore, MICE imputation reduces bias and outputs accurate estimates and variances of associations and we show this by comparing the adjusted differences between the imputed and unimputed datasets (Table B). We observed similar effect estimates but more precise confidence intervals after MICE imputation.

Table B. Adjusted differences in adiposity measures and 95%CI associated per doubling in metal concentrations across various time periods, using multivariable linear regression, comparing imputed and unimputed (complete case) datasets.

	First trimester	Third trimester	Early childhood	Late childhood
A. Female children – Arsenic and zBMI				
MLR (imputed)<br>β (95% CI)	-0.06<br>(-0.24, 0.12)	0.16<br>(0.02, 0.31)	0.00<br>(-0.08, 0.08)	0.05<br>(-0.06, 0.16)
MLR (complete case)<br>β (95% CI)	-0.04<br>(-0.23, 0.14)	0.18<br>(0.02, 0.33)	-0.02<br>(-0.12, 0.09)	0.07<br>(-0.05, 0.18)
B. Female children – Arsenic and zWC				
MLR (imputed)<br>β (95% CI)	-0.01<br>(-0.15, 0.13)	0.13<br>(0.01, 0.25)	-0.03<br>(-0.10, 0.03)	0.06<br>(-0.02, 0.14)
MLR (complete case)<br>β (95% CI)	0.00<br>(-0.14, 0.15)	0.10<br>(-0.02, 0.22)	-0.05<br>(-0.13, 0.03)	0.06<br>(-0.03, 0.16)
